# Editorial: Linked open bibliographic data for real-time research assessment

**DOI:** 10.3389/frma.2023.1275731

**Published:** 2023-09-15

**Authors:** Mohamed Ben Aouicha, Houcemeddine Turki, Mohamed Ali Hadj Taieb

**Affiliations:** Data Engineering and Semantics Research Unit, Faculty of Sciences of Sfax, University of Sfax, Sfax, Tunisia

**Keywords:** bibliographic databases, knowledge graphs, real-time scientometrics, research evaluation, open data, FAIR data, living scientometrics

So far, research evaluation has been very important as a means for deciding academic tenure, awarding research grants, tracking the evolution of scholarly institutions, and assessing doctoral students (King, 1987). However, this effort has been limited by the lack of findability and accessibility of bibliographic databases allowing such an assessment and the legal and financial burdens toward their reuse (Herther, 2009). By the Internet age, scholarly publications became issued online in an electronic format allowing the extraction of accurate bibliographic information from them (Borgman, 2008) as well as the tracking of their readership, download, sharing, and search patterns (Markscheffel, 2013). Online resources called bibliographic knowledge graphs have consequently appeared, providing free bibliographic data and usage statistics for scholarly publications (Markscheffel, 2013). These resources are structured in triples, making them manageable through APIs and query endpoints (Ji et al., 2021). They are kept up-to-date in near real-time through automated methods for enrichment and validation.

Currently, many of these resources are released under permissive licenses such as CC0, CC-BY 4.0, MIT, and GNU covering various aspects of research evaluation (Markscheffel, 2013) including citation data (Peroni and Shotton, 2020), patent information (Verluise et al., 2020), research metadata (Stocker et al., 2022), bibliographic metadata (Hendricks et al., 2020), author information (Haak et al., 2012), and data about scholarly journals, and conferences (Ley, 2009). Multilingual and multidisciplinary open knowledge graphs provide large-scale information about a variety of topics, including bibliographic metadata thanks to user contributions and crowdsourcing within the framework of Linked Open Data (Turki et al., 2022). Due to their flexible data model, they can integrate and centralize knowledge from multiple open and linked bibliographic resources based on persistent identifiers (PID) to become a secondary resource for research data (Nielsen et al., 2017). They also include a large set of non-bibliographic information such as country and prize information that can be used to augment bibliographic data and study the effect of social factors on research efforts (Turki et al., 2022). Later, gathered information can be used to generate research evaluation dashboards that can be updated in real-time based on SPARQL queries (Nielsen et al., 2017) or API queries (Lezhnina et al.). This will allow the launching of a new generation of knowledge-driven living research evaluation (Markscheffel, 2013). Beyond online resources having permissive licenses, several bibliographic databases are available online but have an *All Rights Reserved* license like Google Scholar, maintained by Google (Orduña-Malea et al., 2015), and PubMed, provided by the National Center for Biotechnology Information (Fiorini et al., 2017). These resources can be very useful to feed private research dashboards and real-time research evaluation reports for scholarly institutions.

Despite the value of open bibliographic resources, they can involve inconsistencies that should be solved for better accuracy. As an example, *OpenCitations* mistakenly includes 1,370 self-citations and 1,498 symmetric citations as of April 30, 2022.^1^ As well, they can involve several biases that can provide a distorted mirror of the research efforts across the world (Martín-Martín et al., 2021). That is why these databases need to be enhanced from the perspective of data modeling, data collection, and data reuse. This goes in line with the current perspective of the European Union on reforming research assessment (CoARA, 2022). In this topical collection, we are honored to feature novel research works in the context of allowing the automatic generation of real-time research assessment reports based on open bibliographic resources. We are happy to host research efforts emphasizing the importance of open research data as a basis for transparent and responsible research assessment, assessing the data quality of open resources to be used in real-time research evaluation, and providing implementations of how online databases can be combined to feed dashboards for real-time scholarly assessment.

The four accepted papers in this Research Topic provide insight into the use of open bibliographic data to evaluate academic performance. Majeti et al. present an interface that harvests bibliographic and research funding data from online sources. The authors of this paper address systematic biases in collected data through nominal and normalized metrics and present the results of an evaluation survey taken by senior faculty. Porter and Hook explore the deployment of scientometric data into the hands of practitioners through cloud-based data infrastructures. The authors present an approach that connects Dimensions and World Bank data on Google BigQuery to study international collaboration between countries of different economic classifications. Schnieders et al. evaluate the readiness of research institutions for partially automated research reporting using open, public research information collected via persistent identifiers (PIDs) for organizations (ROR), persons (ORCID), and research outputs (DOI). The authors use internally maintained lists of persons to investigate ORCID coverage in external open data sources and present recommendations for future actions. Lezhnina et al. propose a dashboard using scholarly knowledge graphs to visualize research contributions, combining computer science, graphic design, and human-technology interaction. The user survey showed the dashboard's appeal and potential to enhance scholarly communication through knowledge graph-powered dashboards in different domains.

The research papers featured here underscore the critical importance of open bibliographic data in transforming the landscape of research evaluation. These papers not only shed light on this pivotal role but also offer invaluable practical tools for both researchers and practitioners. By harnessing linked open data, these resources empower individuals within the academic community to navigate the intricacies of scholarly communication more effectively, ultimately leading to improved research assessment practices among scholars and institutions.

## Author contributions

MB: Writing—original draft, Writing—review and editing. HT: Writing—original draft, Writing—review and editing. MH: Writing—original draft, Writing—review and editing.

## Footnotes

^1^A detailed list of deficient self-citations and symmetric citations at OpenCitations can be found at https://github.com/csisc/OCDeficiency.
